# Construction of nursing-sensitive quality indicators for the care of patients with prone position ventilation using the Delphi method

**DOI:** 10.1186/s12912-023-01505-4

**Published:** 2023-09-27

**Authors:** Xiuwen Chen, Peng Liao, Yang Zhou

**Affiliations:** 1 Teaching and Research Section of Clinical Nursing, Xiangya Hospital, Central South University, Changsha, China; 2 National Clinical Research Center for Geriatric Disorders, Xiangya Hospital, Central South University, Changsha, China; 3 Xiangya Nursing School, Central South University, Changsha, China

**Keywords:** Prone position, Ventilation, Nursing, Quality indicators, Delphi Method

## Abstract

**Background:**

Prone position ventilation (PPV) has gradually become an adjuvant treatment to improve oxygenation in patients with acute respiratory distress syndrome. Scientific and comprehensive evaluation of the quality of nursing care for patients with PPV is of great significance to ensure the effectiveness of treatment and patient safety. However, there are no established objective indicators for evaluating the quality of nursing care for patients with PPV. This study intended to identify a set of scientific, systematic and clinically applicable nursing-sensitive quality indicators for the care of patients with PPV.

**Methods:**

Based on the Donabedian structure-process-result theory model, the quality evaluation indicators of nursing care for patients with PPV were preliminarily constructed based on an evidence-based perspective, and two rounds of Delphi surveys were conducted with the purpose of collecting opinions from a panel of independent experts.

**Results:**

The questionnaire recovery rates of the two rounds of correspondence were 100.00% and 95.00%, the recovery rates of expert opinions were 80.00% and 26.32%, the expert authority coefficient values were 0.89, and the Kendall coordination coefficient *W* values were 0.110 and 0.133, respectively. The final nursing-sensitive quality indicators for the care of patients with PPV included 3 first-level indicators, 9 s-level indicators and 29 third-level indicators.

**Conclusion:**

The constructed nursing-sensitive quality indicators for the care of patients with PPV involve quality supervision during the whole process of PPV from three dimensions: structure, process and results. These indicators have strong operability, reliability, practicability and scientificity and can provide a reference for the quality evaluation and monitoring of nursing care for patients with PPV.

**Implications for nursing management:**

The quality indicators of nursing care for patients with PPV constructed in this research are scientific and reliable, and the content of the quality indicators can better reflect the technical characteristics of special nursing. Nursing managers are encouraged to use these quality indicators to evaluate the quality of clinical nursing care and improve safety for patients with PPV.

## Introduction

Prone position ventilation (PPV), in which patients are mechanically ventilated in the prone position, was first developed in the 1970s as a way to treat acute respiratory distress syndrome (ARDS), and proposed as an oxygenation method [1]. PPV benefits patients with ARDS by improving ventilation-perfusion matching, increasing end-expiratory lung volume, and preventing ventilator-induced lung injuries with uniform tidal volume distribution through lung recruitment and alterations in chest wall mechanics [2]. A number of randomized controlled studies have shown that PPV can reduce the pleural pressure gradient in patients, restore ventilation in the dorsal segment of the lung, significantly improve the oxygenation index and blood oxygen saturation in patients, and reduce 28-day mortality risk [3–5]. Especially with the dramatic increase in the number of patients with ARDS caused by the coronavirus disease 2019 (COVID–19) pandemic, PPV is strongly recommended as the main treatment measure for patients with acute respiratory distress and severe and critical COVID–19 [6].

However, PPV, a potentially life-saving adjunctive intervention and an economical, pathophysiological pulmonary protective ventilation strategy, has not been widely used in patients with COVID-19 or ARDS. A survey of ARDS diagnosis and treatment in 50 countries found that the proportion of severe ARDS patients receiving PPV treatment was only 16.3%, and the proportion of severe ARDS patients receiving PPV treatment in China was only 8.7% [7, 8]. Chen et al. [9] showed that the reason why PPV was not widely carried out was related not only to the lack of knowledge, attitudes and behavior of medical staff about PPV but also to the many complications caused by PPV. PPV can increase the incidence of complications such as pressure injuries, periocular injuries, aspiration and arrhythmia [9–12]. A randomized trial of 342 patients found that patients receiving ventilation in a prone position were more likely to experience hypotension or cardiac rhythm disorders (72% vs. 55%), transient oxyhemoglobin desaturation (64% vs. 51%), airway obstruction (51% vs. 34%), vomiting (29% vs. 13%), loss of venous access (16% vs. 4%) and endotracheal tube displacement (11% vs. 5%) than those receiving conventional supine ventilation [13]. Therefore, strengthening the quality management of patients with PPV is expected to reduce the complications related to PPV and improve compliance with PPV treatment.

Nurses are the main practitioners, disease monitors, and caregivers of patients with PPV. However, the measurement of these processes and outcomes is challenging. Nursing-sensitive quality indicators (NSQIs) have been recognized as key measures for evaluating nursing quality and implementing nursing quality improvement measures [14, 15]. NSQIs refer to a set of principles, procedures and evaluation scales used to quantify the level of nursing quality and evaluate nursing effects in clinical nursing practice [16]. Over the years, the quality measurement of nursing care has been developing continuously, which can reflect the effects of nursing measures and their relationships with patient outcomes, thus providing support for clinical decision-making and quality control [17, 18]. At present, the studies on PPV mainly focus on discussing effects, summarizing treatment experiences or sharing nursing points [10, 19]. There are few studies on the evaluation of nursing quality, and there is a lack of a unified standard indicator system for PPV. Therefore, this study intended to build scientific and reasonable NSQIs for prone position ventilation to provide standardized guidance tools for providing nursing care for patients with PPV.

## Methods

### Aim

This study intended to develop a set of scientific, systematic and clinically applicable nursing-sensitive quality indicators for the care of patients with PPV.

### Design

Based on the Donabedian structure-process-result theory model, the quality evaluation indicators of nursing care for patients with PPV were preliminarily constructed based on an evidence-based perspective; 20 experts were consulted, and 2 rounds of Delphi surveys were conducted in May 2022 to establish NSQIs for prone ventilation.

### Literature source and retrieval method

According to the principle of the evidence-based resource 6 S model [20], from top to bottom, the decision support system, evidence thematic summary, guidelines, systematic evaluation and original research related to PPV safety indicators were searched. Researchers searched the PubMed, Embase, Up To Date, Cochrane Library, British Medical Journal (BMJ) best practice, Guidelines International Network, National Institute for Health and Care Excellence, National Guideline Clearinghouse, Scottish Intercollegiate Guidelines Network and Chinese databases (CNKI, Wanfang, medlive.cn) for eligible studies published from 2012 to 2022. Keywords such as prone position, ventilation, respiratory distress syndrome, and quality indicators were chosen. The detailed search strategy for PubMed was as follows.

(((((((prone position[Title]) OR (prone ventilation[Title])) OR (prone positional ventilation[Title])) OR (prone position ventilation[Title])) OR (mechanical ventilation in the prone position[Title])) OR (respiratory distress syndrome[Title])) OR (ARDS[Title]) AND (y_10[Filter])) AND (((((((indicator[Title]) OR (sensitive indicator[Title])) OR (quality indicator[Title])) OR (quality safety[Title])) OR (indicator system[Title])) OR (evaluation indicator[Title])) OR (quality evaluation[Title]) AND (y_10[Filter]))

### Design the letter questionnaire

According to the literature search and quality evaluation results, the definitions, calculation formulas and data collection methods for prone ventilation quality control indicators were extracted, and the evaluation indicators were classified according to the Donabedian structure-process-result three-dimensional quality structure model [21]. Through brainstorming, the research team discussed the applicability and connotation of the indicators, reached a consensus on the names, calculation or collection methods and feasibility of the indicators, and ultimately determined 3 first-level indicators, 9 s-level indicators and 38 third-level indicators to develop the correspondence questionnaire for the first round. The questionnaire consisted of three parts: the preface, indicator consultation form and expert basic information questionnaire. The preface mainly explained the research purpose, significance and matters needing attention. The indicator consultation form mainly included the importance, feasibility and calculation formulas of the indicators, which were scored by experts by using the 5-point Likert scoring method, with columns for “opinions on modifications” and “suggested items to add”. The basic information questionnaire included the experts’ basic information and the degree of expert authority.

### Consultation with experts for selection

In accordance with the principles of academic authority, representativeness and feasibility, a total of 20 experts were selected from 10 comprehensive tertiary-level hospitals, with two experts selected from each hospital. The inclusion criteria of experts were as follows: (1) individuals with a bachelor’s degree or above or an intermediate title or above; (2) individuals with ≥ 10 years of work experience in the field of PPV; (3) individuals with the ability to continue to participate in multiple expert letter consultation rounds until the end of the letter consultation; and (4) individuals who volunteered to participate in the study.

### Implementation of expert letter consultation

The questionnaire was sent and collected by email in May 2022. After the first round of consultation, the expert opinions were summarized, data statistics and analysis were carried out, and indicators with an average importance score < 3.5, an average feasibility score < 3.5 or a coefficient of variation > 0.25 were deleted. The team members focused on revising the questionnaire from the first round of consultations, deleting, modifying or adding indicators. Based on the results for the questionnaire in the first round, the questionnaire for the next round of correspondence was generated.

### Statistical analysis

Two researchers independently inputted the data into Excel tables, and after checking the data correctly, they imported the Excel data into SPSS 22.0 software and then conducted statistical analysis on the data. Descriptive analytical measurement data are expressed as the mean and standard deviation (*SD*), while count data are expressed as the frequency and percentage. The degree of expert participation was expressed by the effective questionnaire recovery rate and the recovery rate of expert opinion submissions. The degree of expert authority was represented by the authority coefficient (*Cr*), which was the mean value of expert familiarity and judgment basis. The degree of expert opinion coordination was expressed by the coefficient of variation (*CV*) and Kendall coordination coefficient *W (Kendall W)*. The significance test was a credibility test of the degree of expert opinion consistency, and the smaller the *P* value was, the higher the credibility of the result. The *P* value was obtained by calculating the *Kendall W* value by SPSS. *P* < 0.05 was considered to be statistically significant.

## Results

### Study selection

**Fig. 1:**
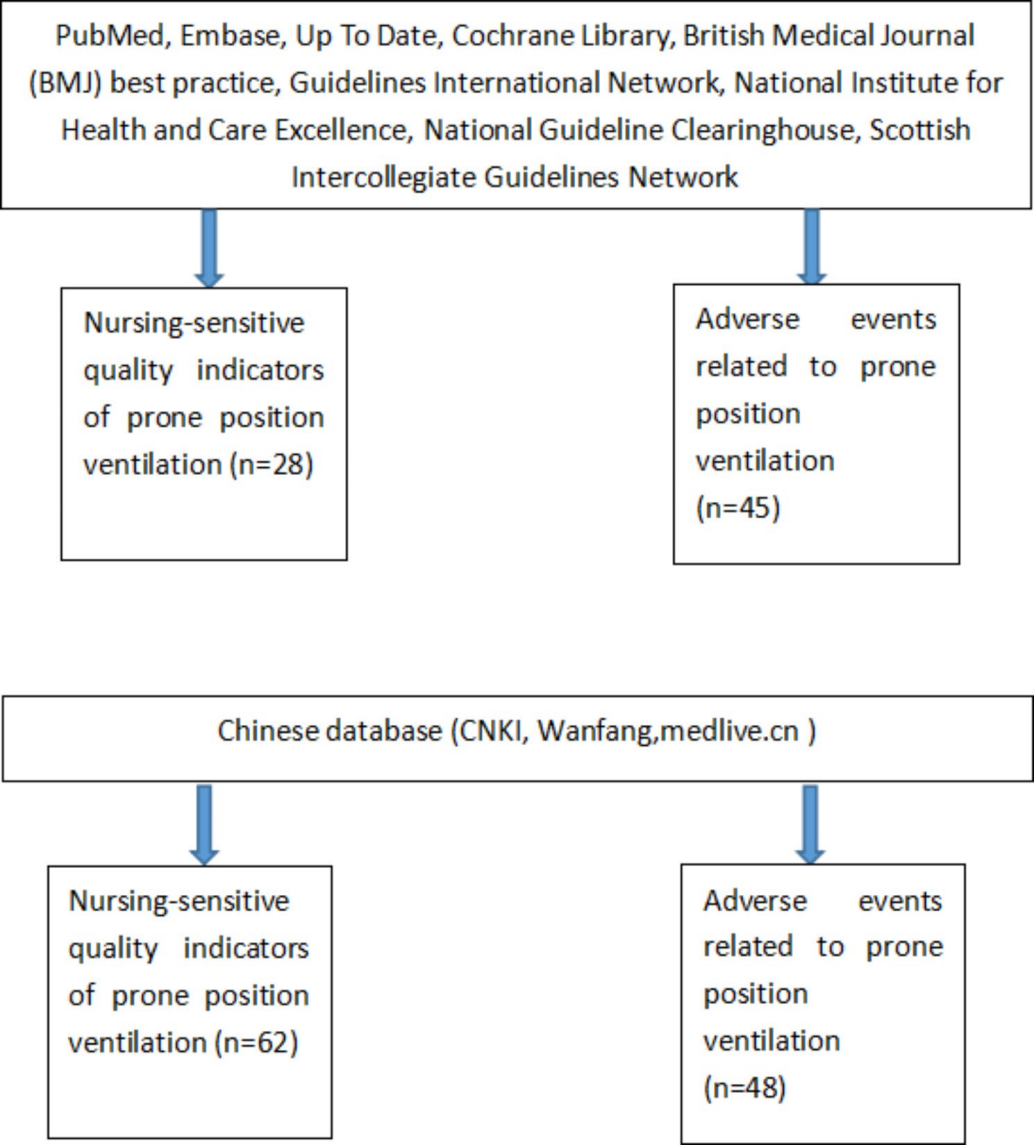
Flow chart of the literature search

A total of 183 articles were retrieved in the initial search. Indicators were mainly derived from the studies as listed (Intensive Care Society 2019 [22], Anika Fourie et al. 2021 [23], Bloomfield R et al. 2015 [24], Atul et al. 2020 [25], Sweet Det al. 2019 [26]). The flow chart of the literature search and retrieval is shown in Fig. 1.

### Basic information of experts

A total of 19 experts from 10 different hospitals were selected for correspondence consultation in this study, including 4 doctors, 3 respiratory therapists and 12 nurses. The age of the experts ranged from 36 to 54 (42.67 ± 4.472) years, and the working experience ranged from 11 ~ 36 (22.00 ± 6.607) years. A total of 2 males (10.53%) and 17 females (89.47%) were included. The degree of education was as follows: 2 experts had doctorate degrees (10.53%), 14 experts had master’s degrees (73.68%), and 3 experts had bachelor’s degrees (15.79%). Regarding professional titles, 10 experts had senior titles (52.63%) and 9 experts had intermediate titles (47.37%). All experts had rich clinical experience in prone ventilation safety management.

### The degree of experts’ activeness, authority and opinion coordination

Two rounds of expert consultation were conducted in this study. In the first round of expert consultation, 20 questionnaires were sent out, and 20 were effectively recovered, with an effective recovery rate of 100.00%. Among these questionnaires, 16 included suggestions for modification, with an expert opinion submission rate of 80.00%. The expert coefficient of judgment was 0.93, the degree of familiarity was 0.84 and *Cr* value was 0.89.

A total of 20 questionnaires were issued for the second round of expert consultation. All the experts consulted were participated in the first round of expert consultation. One expert did not return the questionnaire due to physical discomfort, and 19 questionnaires were effectively recovered, with an effective recovery rate of 95.00%. Five experts put forward suggestions for modification, with a rate of 26.32%. The expert coefficient of judgment was 0.94, the degree of familiarity was 0.84 and the *Cr* value was 0.89. The degree of coordination of the two rounds of expert letter consultation is shown in Table 1.

**Table 1:** The degree of coordination of the two rounds of expert letter consultation

	Importance			Feasibility		
	*Kendall W*	Chi square value	*P value*	*Kendall W*	Chi square value	*P value*
First round(n = 20)	0.110	120.752	0.000	0.115	88.238	0.003
s round(n = 19)	0.133	30.433	0.002	0.088	72.218	0.008

### Results of two rounds of expert letter consultation

#### The first round of expert letter consultation

Three first-level indicators, 9 s-level indicators and 38 third-level indicators were evaluated and statistically analyzed (Table 2). A total of 37 opinions were put forward in the first round of expert consultation. For the ***first-level indicators***, no modifications were suggested. For the ***second-level indicators***, the experts suggested that the order of “resource allocation” in the structural plane should be adjusted to the front of “system and process”. Moreover, the experts proposed that “resource allocation” should be subdivided into “personnel, materials and facilities”. After analyzing and discussing all the expert opinions, the research team believed that the second-level indicators should emphasize the principles of refinement and simplicity and did not agree to make further subdivisions. Finally, the structure indicators in the second-level indicators were divided into “organizational structure”, “resource allocation”, “system process” and “education and training”. There were no suggestions to modify the process and results of the second-level indicators. For the ***third-level indicators***, the experts suggested deleting the following 9 indicators: “performance rate of ventilator parameter evaluation”, “performance rate of lung function evaluation”, “compliance with prone ventilation”, “mechanical ventilation duration”, “qualified rate of nursing records”, “patient comfort level”, “incidence of total complications of prone ventilation”, “incidence of facial edema”, and “incidence of peripheral nerve injuries”. The experts suggested revising 6 third-level indicators. The following suggestions were made: “the number of operators” and “the responsibilities of each operator role” be merged into “requirements of operators”; “fully prepared materials” be modified to “material readiness rate”; “the completion rate of monitoring facilities” and “the completion rate of emergency facilities” be merged into “first aid and monitoring facilities completion rate”; “the implementation rate of respiratory/airway preparation” be modified to “the implementation rate of airway preparation”; “implementation rate of sedation state assessment” be revised to “execution rate of sedation and analgesia assessment”; and “prone position ventilation duration” be revised to “standard prone position ventilation duration rate”. Two new third-level indicators were added, namely, the incidence of unplanned treatment interruptions in the prone position and the incidence of eye injuries.

In addition, the importance scores of each indicator from the first round of expert consultation ranged from 4.45 to 5.00, and the *CV* value ranged from 0.00 to 0.23. The feasibility scores ranged from 3.94 to 4.90, and the *CV* value ranged from 0.06 to 0.24. Therefore, there was no need to delete the indicators due to the statistical analysis. Ultimately, 3 first-level indicators, 9 s-level indicators and 29 third-level indicators were selected to form the questionnaire for the second round of expert correspondence.

**Table 2:** The results of the first round of expert consultation

First-level indicators	Second-level indicators	Third-level indicators		Importance			Applicability of data collection methods	
		Indicators	Calculation formula	Mean(*SD*)	*CV*	Weight(%)	Mean(*SD*)	*CV*
Structure indicators	Organizational Structure	1. Setup of the PPV management team	A or B/1 × 100%, where A = 1, indicating that there is a PPV management team; B = 0, indicating that there is no PPV management team	4.50 ± 0.827	0.18	0.025	4.70 ± 0.657	0.14
	System and Process	2. Emergency plan system for security incidents	A or B/1 × 100%, where A = 1, indicating that there is a security incident emergency plan system; B = 0: there is no emergency plan system for security incidents	4.70 ± 0.571	0.12	0.026	4.53 ± 0.905	0.21
		3. PPV operation procedure	A or B/1 × 100%, where A = 1, indicating that a PPV operation procedure has been developed; B = 0, indicating that there is no PPV operation procedure	4.90 ± 0.308	0.06	0.027	4.89 ± 0.315	0.06
		4. Checklist of PPV operations	A or B/1 × 100%, where A = 1, indicating that there is a checklist of PPV operations; B = 0, indicating that there is no checklist of PPV operations	4.80 ± 0.523	0.11	0.027	4.74 ± 0.562	0.12
	Resource Allocation	5. Number of operators	Total number of medical personnel able to perform PPV	4.55 ± 0.605	0.13	0.025	4.68 ± 0.582	0.12
		6. Responsibilities of each operator role	A or B/1 × 100%, where A = 1, indicating clear responsibilities of each operator role; B = 0: The responsibilities of each role are not clearly defined	4.65 ± 0.933	0.20	0.026	4.50 ± 0.607	0.13
		7. Fully prepared materials	A or B/1 × 100%, where A = 1, materials are well prepared before performing PPV; B = 0: The materials are not well prepared before performing PPV	4.85 ± 0.366	0.08	0.027	4.60 ± 0.821	0.18
		8. The completion rate of monitoring facilities	Times of completion for monitoring facilities/the total number of random checks within the cycle ×100%	4.75 ± 0.550	0.12	0.026	4.85 ± 0.366	0.08
		9. The completion rate of emergency facilities	Times of completion for emergency facilities/the total number of random checks within the cycle ×100%	4.80 ± 0.523	0.11	0.027	4.45 ± 0.887	0.20
	Education and Training	10. Implementation rate of PPV-related training	Actual times of PPV training/total planned training times of the same period ×100%	4.85 ± 0.366	0.08	0.027	4.70 ± 0.571	0.12
		11. Qualified rate of knowledge assessment of PPV	Person-times qualified for PPV knowledge examinations/total person-times of spot checks ×100%	4.65 ± 0.671	0.14	0.026	4.55 ± 0.686	0.15
Process indicators	Before switching positions	12. Performance rate of hemodynamic assessment	Person-times of hemodynamic assessment/total person-times of spot checks within the cycle ×100%	4.95 ± 0.308	0.06	0.028	4.75 ± 0.786	0.17
		13. The implementation rate of respiratory or airway preparation	Person-times of respiratory or airway preparation/total number of spot checks performed during the cycle ×100%	5.00 ± 0.000	0.00	0.028	4.79 ± 0.419	0.09
		14. Implementation rate of sedation state assessment	The number of people who performed sedation assessment/the total number of random checks within the cycle ×100%	4.90 ± 0.308	0.06	0.027	4.85 ± 0.489	0.10
		15. Implementation rate of gastric residual volume assessment	Person-times of gastric residual volume assessment/total person-times of spot checks ×100%	4.84 ± 0.375	0.08	0.027	4.70 ± 0.657	0.14
		16. Implementation rate of pressure injuries risk assessment	Person-times of pressure injuries risk assessments/total person-times of spot checks ×100%	4.75 ± 0.550	0.12	0.026	4.80 ± 0.523	0.11
		17. Execution rate of unplanned extubation risk assessment	Person-times of unplanned extubation risk assessments/total person-times of spot checks ×100%	4.90 ± 0.308	0.06	0.027	4.35 ± 0.933	0.21
		18. Timeout execution rate	Person-times of timeout execution/total person-times of spot checks within the cycle ×100%	4.90 ± 0.308	0.06	0.027	4.85 ± 0.489	0.10
		19. Performance rate of ventilator parameter evaluation	Person-times of ventilator parameter evaluation/total person-times of spot checks ×100%	4.75 ± 0.639	0.13	0.026	4.55 ± 0.887	0.19
	While changing position	20. Performance rate of dynamic observation during postural transition	Number of patients undergoing dynamic observation during position changes/total number of spot checks ×100%	4.95 ± 0.224	0.05	0.027	4.84 ± 0.375	0.08
		21. Implementation rate of the checklist	Number of implementation entries on the checklist/total entries on the checklist within the period ×100%	4.74 ± 0.452	0.10	0.026	4.85 ± 0.489	0.10
	After changing position	22. Standard position placement rate	Number of patients with qualified position placement within the cycle/total number of persons with spot checks ×100%	4.55 ± 0.887	0.19	0.025	4.35 ± 0.998	0.23
		23. Airway assessment execution rate	Times of airway assessment after postural change/total number of spot checks within the cycle ×100%	4.80 ± 0.696	0.15	0.027	4.74 ± 0.653	0.14
		24. Performance rate of lung function evaluation	Number of lung function evaluations after a postural change/total number of spot checks within the cycle ×100%	4.55 ± 0.887	0.19	0.025	4.35 ± 0.988	0.23
		25. Compliance with prone ventilation	Number of patients with PPV/total number of patients requiring PPV ×100%	4.65 ± 0.587	0.13	0.026	4.33 ± 0.907	0.21
Result indicators	Quality of nursing care	26. Prone position ventilation duration	Total PPV hours per patient	4.75 ± 0.639	0.13	0.026	4.74 ± 0.653	0.13
		27. Oxygenation index improvement rate	Number of patients with an improved oxygenation index after PPV/total number of patients with PPV ×100%; Oxygenation index = arterial oxygen partial pressure (PaO2)/oxygen absorption concentration (FiO2) ×100%	4.55 ± 0.826	0.18	0.025	4.58 ± 0.838	0.18
		28. Mechanical ventilation duration	Total hours of mechanical ventilation per patient	4.50 ± 1.000	0.22	0.025	4.42 ± 0.838	0.19
		29. Qualified rate of nursing records	Number of qualified nursing records/total number of spot checks ×100%	4.45 ± 0.887	0.20	0.025	4.79 ± 0.419	0.09
		30. Lung reexpansion rate	Patients with recurrent lung expansion after PPV/total patients with PPV ×100%	4.80 ± 0.410	0.09	0.027	4.56 ± 0.616	0.14
		31. Patient comfort level	Patient comfort scores for awake prone patients	4.60 ± 0.681	0.15	0.026	4.58 ± 0.507	0.11
	Adverse Events	32. Incidence of total complications of prone ventilation	Number of patients with complications due to PPV/total patients with PPV in the same period × 100%	4.75 ± 0.550	0.12	0.026	4.04 ± 0.629	0.16
		33. Incidence of pressure injuries	Number of patients with pressure injuries/total patients with PPV in the same period × 100%	4.90 ± 0.308	0.06	0.027	4.75 ± 0.716	0.15
		34. Incidence of unplanned extubation	Person-times of unplanned extubation/Total person-times of PPV in the same period × 100%	4.85 ± 0.489	0.10	0.027	4.39 ± 1.000	0.23
		35. Incidence of aspiration/vomiting	Number of patients with aspiration/vomiting/total patients with PPV × 100%	4.45 ± 0.887	0.20	0.025	4.60 ± 0.940	0.20
		36. Incidence of facial edema	Number of patients with facial edema due to PPV/total patients with PPV in the same period × 100%	4.65 ± 0.813	0.17	0.026	4.50 ± 0.761	0.17
		37. Incidence of bed falls	Number of patients falling out of bed/total patients with PPV in the same period × 100%	4.55 ± 0.826	0.06	0.025	4.75 ± 0.550	0.12
		38. Incidence of peripheral nerve injuries	Number of patients with peripheral nerve injuries due to PPV/total patients with PPV in the same period × 100%	4.45 ± 0.887	0.20	0.025	4.20 ± 0.967	0.23

Note: *SD, standard deviation;CV, coefficient of variation; PPV, prone position ventilation*

#### The second round of expert letter consultation

In the second round of expert consultation, a total of six suggestions were put forward. There were no suggestions to modify the first-level and second-level indicators. For the third-level indicators, the experts suggested that “oxygenation index” be revised to “oxygenation index improvement rate”; the “performance rate of hemodynamic assessment” was changed to “implementation rate of blood pressure evaluation”. However, after consulting the literature and group discussion, it was considered that not only blood pressure but also blood flow, blood flow resistance and their interrelationship should be evaluated before position conversion to prevent the occurrence of cardiac arrhythmia, heart failure and other complications; the opinion was not adopted. “Incidence of aspiration/vomiting” was suggested to be changed to “incidence of reflux/aspiration”. After brainstorming and discussion, it was considered that the collection feasibility of “reflux” was poor, so the opinion was not adopted. In addition, one-on-one interviews with experts were conducted to explain the reasons for not accepting the opinions and to obtain their consent.

The importance scores of each index in the second round of expert letter consultation ranged from 4.47 to 5.00, and the *CV* value ranged from 0.00 to 0.23. The feasibility scores ranged from 4.47 to 5.00, and the *CV* value ranged from 0.00 to 0.24. Therefore, there was no need to delete indicators due to the statistical analysis. After sorting, analyzing and summarizing the expert letter consultation results, the research team ultimately established the NSQIs for prone position ventilation, which included 3 first-level indicators, 9 s-level indicators and 29 third-level indicators, as shown in Table 3.

**Table 3:** Nursing-sensitive quality indicators for prone position ventilation

First-level indicators	Second-level indicators	Third-level indicators		Importance			Applicability of data collection methods	
		Indicators	Calculation formula	Mean(*SD*)	*CV*	Weight(%)	Mean(*SD*)	*CV*
Structure indicators	Organizational Structure	1. Setup of the PPV management team	A or B/1 × 100%, where A = 1, indicating that there is a PPV management team; B = 0, indicating that there is no PPV management team	4.74 ± 0.452	0.10	0.029	4.95 ± 0.229	0.05
	Resource Allocation	2. Requirements of the operators	A or B/1 × 100%, where A = 1, indicating clear roles, responsibilities and requirements of the operators; B = 0: The roles, responsibilities and requirements of operators are not clearly defined	4.74 ± 0.452	0.10	0.029	4.79 ± 0.535	0.11
		3. Material readiness rate	Number of times that the materials were ready before implementing PPV/total number of spot checks ×100%	4.84 ± 0.501	0.10	0.029	4.89 ± 0.315	0.06
		4. First aid and monitoring facilities completion rate	Times of complete first aid and monitoring facilities/total number of spot checks within the cycle ×100%	4.84 ± 0.501	0.10	0.029	4.89 ± 0.315	0.06
	System and Process	5. Emergency plan system for security incidents	A or B/1 × 100%, where A = 1, indicates that there is a security incident emergency plan system; and B = 0 indicates that there is no emergency plan system for security incidents	4.74 ± 0.452	0.10	0.029	4.95 ± 0.229	0.05
		6. PPV operation procedure	A or B/1 × 100%, where A = 1, indicating that a PPV operation procedure has been developed; B = 0, indicating that there is no PPV operation procedure	4.89 ± 0.315	0.06	0.030	5.00 ± 0.000	0.00
		7. Checklist of PPV operations	A or B/1 × 100%, where A = 1, indicating that there is a checklist of PPV operations; B = 0, indicating that there is no checklist of PPV operations	4.84 ± 0.375	0.08	0.029	4.84 ± 0.375	0.08
	Education and Training	8. Implementation rate of PPV related training	Actual times of PPV training/total planned training times in the same period ×100%	4.74 ± 0.452	0.10	0.029	4.79 ± 0.419	0.09
		9. Qualified rate of knowledge assessment of PPV	Person-times qualified in PPV knowledge examination/total person-times of spot checks ×100%	4.68 ± 0.582	0.12	0.029	4.79 ± 0.419	0.09
Process indicators	Before switching positions	10. Performance rate of hemodynamic assessment	Person-times of hemodynamic assessment/total person-times of spot checks within the cycle ×100%	4.79 ± 0.535	0.11	0.029	4.68 ± 0.582	0.12
		11. Airway preparation performance rate	Person-times of airway preparation/total number of spot checks performed during the cycle ×100%	4.95 ± 0.229	0.05	0.030	4.95 ± 0.229	0.05
		12. Execution rate of sedation and analgesia assessment	Number of people who performed sedation and analgesia assessments/total number of spot checks within the cycle ×100%	4.84 ± 0.375	0.08	0.029	4.79 ± 0.419	0.09
		13. Implementation rate of gastric residual volume assessment	Person-times of gastric residual volume assessments/total person-times of spot checks ×100%	4.84 ± 0.375	0.08	0.029	4.68 ± 0.582	0.12
		14. Implementation rate of pressure injuries risk assessment	Person-times of pressure injuries risk assessments/total person-times of spot checks ×100%	4.89 ± 0.315	0.06	0.030	4.89 ± 0.315	0.06
		15. Execution rate of unplanned extubation risk assessment	Person-times of unplanned extubation risk assessments/total person-times of spot checks ×100%	4.89 ± 0.315	0.06	0.030	4.89 ± 0.315	0.06
		16. Timeout Execution rate	Person-times of timeout execution/total person-times of spot checks within the cycle ×100%	4.89 ± 0.315	0.06	0.030	4.89 ± 0.315	0.06
	While changing position	17. Performance rate of dynamic observation during postural transition	Number of patients undergoing dynamic observation during position changes/total number of spot checks ×100%	5.00 ± 0.000	0.00	0.030	4.63 ± 0.597	0.13
		18. Implementation rate of checklist	Entries/total entries on the checklist within the period ×100%	4.74 ± 0.452	0.10	0.029	4.47 ± 0.697	0.16
	After changing position	19. Standard position placement rate	Number of persons with qualified position placement within the cycle/total number of persons with spot checks ×100%	5.00 ± 0.000	0.00	0.030	4.89 ± 0.315	0.06
		20. Airway assessment execution rate	Airway assessment after postural changes/total number of spot checks within the cycle ×100%	4.89 ± 0.315	0.06	0.030	4.84 ± 0.375	0.08
Result indicators	Quality of nursing care	21. PPV duration standard rate	Number of patients with qualified PPV duration/total number of patients with PPV duration ×100%	5.00 ± 0.000	0.00	0.030	4.63 ± 0.761	0.16
		22. Incidence of unplanned treatment interruptions in PPV	Patients with interruptions of unplanned PPV treatment/total number of patients with PPV ×100%	4.63 ± 0.597	0.13	0.028	4.53 ± 0.697	0.15
		23. Oxygenation index improvement rate	Number of patients with an improved oxygenation index after PPV/total number of patients with PPV ×100%; Oxygenation index = arterial oxygen partial pressure (PaO2)/oxygen absorption concentration (FiO2) ×100%	4.79 ± 0.535	0.11	0.029	4.63 ± 0.684	0.15
		24. Lung reexpansion rate	Number of patients with recurrent lung expansion after PPV/total number of patients with PPV ×100%	4.80 ± 0.410	0.09	0.029	4.79 ± 0.419	0.09
	Adverse Events	25. Incidence of pressure injuries	Number of patients with pressure injuries/total number of patients with PPV in the same period × 100%	4.89 ± 0.315	0.06	0.030	4.89 ± 0.315	0.06
		26. Incidence of unplanned extubations	Number of patients with unplanned extubations/total number of patients with PPV in the same period × 100%	5.00 ± 0.000	0.00	0.030	4.95 ± 0.229	0.05
		27. Incidence of aspiration/vomiting	Number of patients with aspiration/vomiting/total number of patients with PPV in the same period × 100%	4.74 ± 0.562	0.12	0.029	4.89 ± 0.459	0.09
		28. Incidence of eye injuries	The number of patients with eye injuries/total number of patients with PPV in the same period × 100%	4.84 ± 0.375	0.08	0.029	4.68 ± 0.671	0.14
		29. Incidence of bed falls	Number of patients falling out of bed/total number of patients with PPV in the same period × 100%	4.68 ± 0.582	0.12	0.029	4.63 ± 0.684	0.15

Note: *SD, standard deviation;CV, coefficient of variation; PPV, prone position ventilation*

## Discussion

### The NSQIs for prone position ventilation are scientific and reliable

It is urgent to develop unified NSQIs for prone position ventilation and develop a nursing quality evaluation system to make nursing quality evaluation more accurate and efficient. In this study, based on the Donabedian structure-process-result theory model, the NSQIs of care for patients with PPV were initially constructed using an evidence-based perspective, and then the importance of each index was scored based on the Delphi method. The scientific rigor of the research method promoted the reliability of these research results. In expert correspondence consultation, it is generally believed that the effective questionnaire recovery rate is more than 70.00% [27]. In this study, the effective questionnaire recovery rate of the two rounds of consultations was far greater than this value, indicating that experts had high enthusiasm for this study. Research has shown that a degree of expert authority > 0.70 is acceptable [17]. The degree of authority of the two rounds of expert consultation was much higher than the acceptable value, indicating that experts had high authority. The result of *Kendall W* values also indicated that the degree of coordination of expert opinions was great and that the correspondence consultation was reliable. In addition, the selection of experts is crucial to the Delphi method. The experts selected in this study included not only nurses but also ICU doctors and respiratory therapists, who all had rich experience in the implementation of prone position ventilation. All the above findings indicate that the NSQIs for the care of patients with PPV in this study are scientific and reliable, and nursing managers are encouraged to use these quality indicators to evaluate the quality of clinical nursing and improve the safety of PPV.

### The content of NSQIs for prone position ventilation can better reflect the special nursing technical characteristics

#### The structural indicators mainly include organizational structure, resource allocation, system and process, and education and training

Structural indicators refer to the organizational, institutional, human resource, configuration and other structural factors that can affect the quality of medical care in medical institutions [11]. After two rounds of consultation, the study determined that the structural indicators comprised four second-level indicators, namely, organizational structure, resource allocation, system process and education and training, and 9 third-level indicators. Reasonable setup of the PPV management team, standard PPV procedures, and adequate materials, equipment, and emergency plan systems for security incidents are prerequisites for ensuring the quality of PPV care and patient safety. As recommended in a summary of the evidence for PPV in adults with ARDS published in the Up to Date database in 2022 [25], PPV management teams should include physicians, respiratory therapists, and nurses. It also pointed out that adequate quality monitoring and training of the prone ventilation management team is very important to improve the quality of prone ventilation care and patient safety. A qualitative study on prone ventilation by Yang Jing et al. [28] also showed that the implementation and promotion of prone ventilation were seriously affected by insufficient allocation of human resources, unclear procedures and programs, and insufficient awareness of prone ventilation among medical staff. Second, the results of this study showed that the PPV operation procedure accounted for the largest proportion of indicators among the 9 third-level structural indicators. This indicated that medical institutions and managers should develop standardized prone position ventilation operation procedures to promote the homogeneity of prone position treatment. At present, although a large number of research results on the effect of prone ventilation have been published, there is still no systematic standard operating process and program to provide clinical guidance, which is a major blind spot for medical staff. Therefore, the prone ventilation operation process and the prone ventilation operation checklist were unanimously recognized by experts as structural indicators of quality control in this study. In addition, two educational and training indicators, namely, the implementation rate of PPV-related training and the qualified rate of knowledge assessment of PPV, were included as structural indicators. Education and training is an important guarantee to promote the ventilation quality of the prone position in patients with ARDS or COVID-19, as well as an important measure to improve the cognition level of medical staff regarding PPV, reduce the occurrence of PPV-related complications and improve the utilization rate of PPV. The content of education and training in this study included the principle, indications, operation process, complication prevention and risk emergency plan of prone ventilation.

#### The process indicators focused on nursing evaluation and nursing measures

Process indicators are the core of the prone ventilation quality control index, which can comprehensively reflect the nursing care quality and safety for patients with prone ventilation. In this study, 11 process indicators were developed for the three steps of prone position ventilation: before, during and after position conversion. Quality control of the nursing process is conducted from several aspects, including hemodynamic assessment, airway preparation, sedation and analgesia assessment, gastric residual volume assessment, pressure injuries risk assessment, unplanned extubation risk assessment, timeout execution, dynamic observation during postural transitions, checklist execution, posture placement and airway assessment. These can reflect the implementation of prone ventilation and quality control results. An expert consensus showed that hemodynamic assessment should be emphasized when standardizing overall care measures for patients with severe and critical COVID-19. Airway preparation mainly includes the ventilator being as close to the patient’s side as possible, the difficult airway intubation vehicle and negative pressure suction being in a standby state, rechecking the patient’s laryngoscope and the length of the tracheal tube, fixing or binding the tracheal catheter, oxygenating the patient with 100% oxygen, monitoring the tidal volume and inspiratory pressure, etc. Atul [25] suggested that a respiratory therapist ensure the stability of the endotracheal tube during the whole process of position transitions. In this study, the risk assessment of process indicators mainly included sedation and analgesia assessment, gastric residual volume assessment, stress injuries risk assessment and unplanned extubation risk assessment. The application of effective risk assessment tools to accurately assess the risk factors patients is the premise of ensuring patient safety. The Richmond Agitation-Sedation Scale (RASS) [29], Braden Scale for Pressure Ulcer Risk [30] and Catheter Risk Scale [31] are recommended for risk assessment before prone ventilation. The guidelines [22] suggested that after everything is prepared, timeout should be activated before position conversion to help reduce the occurrence of complications and other adverse events. Dynamic observation of the patient’s condition is very important in changing positions, and changes in blood pressure, heart rate and respiration should be monitored. The standard rate of postural position after transition is mainly evaluated from the perspective of postural position and pipe management. For patients with PPV, their limbs should be placed in a functional position throughout the whole process to prevent pain and other discomfort caused by improper positioning, which will affect the ventilation duration and treatment effect of prone position ventilation.

#### The outcome indicators focused on the quality of nursing care and adverse events

The result indicators are the comprehensive embodiment of the structural plane and process plane and can also provide feedback control of the quality of the structural plane and process plane. The results of this study identified three secondary indicators of quality of medical care and safety incidents and 9 third-level indicators. The quality of nursing care includes the standard rate of ventilation duration in the prone position, the interruption rate of treatment in the unplanned prone position, the improvement rate of the oxygenation index and the lung re-expansion rate, among which the ventilation duration in the prone position has the highest weight among the outcome indicators. The 2019 international Guidelines [26] Official Guidelines: *European Consensus Guidelines on the Management of Respiratory Distress Syndrome − 2019 Update* strongly recommend prone ventilation for at least 16 h in ARDS patients with an oxygenation index less than 150 mmHg, suggesting that this index should be the focus of quality evaluation. The oxygenation index and pulmonary reexpansion are effective evaluation indices of prone ventilation, which can provide a quantifiable basis for medical care quality and curative effect analysis. The results of this study showed that the main adverse events in the result indicators were common complications of prone ventilation. Studies have shown that prone ventilation also includes brachial plexus injuries, crush injuries, arrhythmia and other complications [25]. However, this study adhered to the characteristics of “few but fine” quality indicators and did not include all of them. In clinical operation management, medical institutions can choose indicators to guide nursing quality management according to the characteristics of complications, improve the awareness of prone ventilation safety management, avoid risk factors, and reduce adverse outcomes.

## Limitations

This study had several limitations. First, this study was performed in the context of the Chinese health care system. Our inferences may not necessarily be relevant for patients in other parts of the world. Second, in Delphi expert consultation, the selection of experts is crucial, and the experts selected in this study were from only ten hospitals. It still cannot represent the whole situation of the whole country because of the enormous area of China. The views of the included Delphi panelists may also differ from those of experts who did not participate. To try to minimize this limitation, a comprehensive search can be conducted among more experts from more hospitals. Third, this was a preliminary study to develop potential NSQIs for the evaluation of the quality of nursing care for patients with PPV. Their applicability needs further investigation after applying them in clinical practice.

## Conclusion

In this study, based on the Donabedian structure-process-result model as the theoretical basis and on the basis of previous research and practice, current situation analysis and domestic and foreign literature retrieval, the Delphi method was applied using an evidence-based perspective to construct NSQIs for prone position ventilation, including 3 first-level indicators, 9 s-level indicators and 29 third-level indicators. Based on the premise of improving nursing management and patient outcomes, the indicators cover the key contents of prone ventilation safety evaluation. These indicators are scientific, objective, reliable and operable and can provide guidance for quality control. Due to time constraints, the indicator system was only tested in the intensive care unit of a designated hospital for the treatment of COVID-19 in Shanghai and has not been tested in a multicenter clinical trial. The feasibility, applicability and sensitivity of the indicators need to be further confirmed. Future research will develop in this direction, continue to carry out prone ventilation quality management under normal epidemic prevention and control, and develop continuous improvement strategies for nursing quality according to the results, further improving the content of indicators and patient safety.

## Data Availability

All data generated or analysed during this study are included in this published article.
